# Effect of Annealing on the Mechanical Properties of Carbon Fiber-Reinforced PLA

**DOI:** 10.3390/polym18212721

**Published:** 2026-11-02

**Authors:** Daniel Perez-Solis, Jose Martinez-Trinidad, Alvaro Frutos, Manuel Coca-Gonzalez, Moises Jimenez-Martinez

**Affiliations:** 1 Grupo Ingeniería de Superficies, ESIME Zacatenco, Instituto Politecnico Nacional, Mexico City C.P. 07738, Mexico; dperezs1802@alumno.ipn.mx (D.P.-S.); jomartinezt@ipn.mx (J.M.-T.); 2 Kautex Textron de Mexico, S. de R.L. de C.V. Autop., Heroica Puebla de Zaragoza C.P. 72761, Mexico; josealvaro.frutos@kautex.com; 3 School of Engineering and Sciences, Tecnologico de Monterrey, Via Atlixcayotl 5718, Puebla C.P. 72453, Mexico; A01666072@tec.mx

**Keywords:** 3D printing, annealing, Response Surface Methodology, mechanical properties

## Abstract

Additive manufacturing of polylactic acid reinforced with carbon fiber, specifically via fused deposition modeling, offers significant advantages for fabricating complex geometries; however, inherent extrusion defects often restrict its use in high-performance mechanical components. While post-printing annealing treatments promote polymer crystallization and interlayer cohesion, the highly non-linear effects of this process on PLA composites remain underexplored. This study investigates the specific effects of annealing temperature and exposure time on parametrically optimized 3D-printed polylactic acid reinforced with short carbon fiber. An orthogonal experimental design evaluates temperatures ranging from 55 °C to 95 °C and times between 4 and 12 h. The mechanical behavior was mapped utilizing Analysis of Variance and Response Surface Methodology, supported by Differential Scanning Calorimetry and dimensional analysis. The results revealed that annealing temperature is the governing factor, whereas exposure time has a marginal impact. An optimal window emerged between 75 °C and 85 °C. Relative to the as-printed baseline, optimal factors improved Ultimate Tensile Strength by 13.74%, ductility by 24.78%, bed surface hardness by 5.58% and Young’s modulus by 39%, driven by substantial matrix crystallinity increments (29.4%) verified via DSC. In contrast, top surface hardness was negatively affected by the heat treatment. Furthermore, temperatures exceeding 85 °C triggered severe dimensional distortions. Ultimately, this research provides experimentally validated statistical models for the reliable design of PLA-SCF components.

## 1. Introduction

Additive Manufacturing (AM) technologies have profoundly transformed modern engineering by overcoming the limitations of traditional manufacturing methods, enabling the direct fabrication of three-dimensional objects with highly complex geometries from computer-aided models [1]. The significance of AM spans across diverse application areas, offering critical advantages such as rapid prototyping, the creation of lightweight structural components, and highly customized designs. Based on the general classification of AM processes, Fused Deposition Modeling (FDM), often referred to as Fused Filament Fabrication (FFF), is categorized as a material extrusion technology. FDM has become one of the most widely adopted methods globally due to its low equipment cost, broad accessibility, and compatibility with a wide range of thermoplastic materials [2].

The FDM process fundamentally consists of continuously feeding a thermoplastic filament into a heated nozzle, where it is melted and deposited layer by layer onto a build platform to form the final component. Among the available thermoplastic matrices, Polylactic Acid (PLA) is predominantly utilized owing to its biodegradability, affordability, and ease of processing. Nevertheless, the intrinsic defects generated during the FDM process, such as interlaminar debonding, micro-voids, and severe anisotropy, restrict the application of these materials in high-performance structural components [3,4].

To overcome these limitations, studies have focused on incorporating reinforcement agents, with carbon fibers proving highly effective in engineering applications. Wang et al. [5] demonstrated that an optimal 7 wt% short carbon fiber (SCF) fraction enhances mechanical properties, as the fibers distribute stresses in the polymer matrix.

Furthermore, optimizing the 3D printing parameters is a crucial step to maximize this material’s mechanical performance, which has demonstrated to enhance the Ultimate Tensile Strength (UTS) by more than 30% [6]. For PLA-SCF composites, printing speed and layer thickness are exceptionally influential [7]. Lower printing speeds allow the short carbon fibers sufficient time to homogeneously organize and reorient along the printing path, redirecting parallel to the tensile load and successfully transferring it to the matrix [8]. Simultaneously, minimizing layer thickness enhances the thermal diffusion of polymer chains between adjacent filaments, strengthening the cohesive force between layers [9,10].

However, even with optimally tuned printing parameters, FDM inherently leaves residual stresses due to heat gradients [11] and limits the maximum achievable interlaminar strength. Consequently, post-printing thermal treatments have emerged as a fundamental requirement to further increase the mechanical performance.

Annealing is the most prominent of these treatments [12]; supplying heat energy to the polymer chains above the Glass Transition Temperature ($T_{g}$) enhances interlayer bonding by facilitating their diffusion across printed boundaries [13] and promotes the crystallization of the matrix by generating highly organized atomic regions with superior cohesive energy. These microstructural changes directly improve the UTS, impact strength, and hardness and typically reduce ductility [14,15].

Nevertheless, annealing benefits do not necessarily evolve linearly with temperature or time and may affect each mechanical property differently depending on the initial as-printed microstructure. Therefore, identifying an optimal annealing window is critical, since excessive thermal exposure can shift the outcome from microstructural enhancement to thermal degradation, even when crystallinity continues to increase [16,17].

While neat PLA has a well-documented optimal annealing window [18,19], thermal treatments for PLA composites remain underexplored. Recent investigations suggest an optimal region typically between 70 °C and 90 °C [20]. Within this range, annealing temperature acts as the dominant driving factor, whereas exposure time has proven to be statistically non-significant for PLA-CF [4,21,22]. This lack of time dependency has opened a promising research window focusing on extremely short exposure times at elevated temperatures (100 °C to 110 °C) [23]. However, the current literature warns that exceeding these thermal limits requires rigorous monitoring to prevent the rapid onset of material degradation and dimensional distortion [24,25].

Despite the growing interest in post-printing thermal treatments, the existing literature predominantly focuses on isolated mechanical properties using non-optimized as-printed baselines. This approach hides the true benefits of annealing with the mere correction of poor printing parameters. Previous studies lack a coupled analysis that simultaneously evaluates mechanical enhancement, microstructural evolution, and the inevitable dimensional distortion which is a critical barrier for practical engineering applications. Comprehensive behavioral mapping using robust statistical models that can predict these combined effects across a broad spectrum of annealing parameters remains notably absent.

To bridge this gap, this study isolates the thermomechanical effects of annealing by utilizing a parametrically optimized 3D-printed PLA-SCF baseline. The primary contribution of this work is the development of experimentally validated statistical models (ANOVA and RSM) that comprehensively map the evolution of UTS, Young’s modulus, ductility, and localized hardness across different annealing temperatures and exposure times. By coupling these mechanical maps with DSC microstructural analysis and dimensional distortion quantification, this study provides an engineering tool to identify the practical annealing window where mechanical performance is maximized without critically compromising the geometric integrity of the component.

## 2. Materials and Methods

### 2.1. Filament

A commercial black PLA-CF filament of BambuLab, with a 1.75 mm diameter and a short carbon fiber content of 6.88 w%, was utilized to fabricate all specimens in this study [26]. The optimal parameters and conditions for the printing process are detailed in Table 1 based on [6]. The part cooling fan during printing was set to 100% capacity in accordance with the recommendations from the manufacturer, followed by an ambient cooling directly on the build plate.

The corresponding material properties are summarized in Table 2. In this context, the non-optimized printing parameters refer strictly to the baseline data and standard deviations reported in the BambuLab technical data sheet, which reflect the material’s performance using default printing profiles. Conversely, the optimized printing parameters correspond to the enhanced mechanical responses achieved in the previous study [6] where a batch of three specimens per experimental level was evaluated. The only exception was Young’s modulus, which in this investigation was determined with a larger batch of five specimens to precisely determine the final optimized property.

Thermal properties are reported in the manufacturer’s data sheet where $T_{g}$ and melting temperature ($T_{m}$) are established at 63 °C and 165 °C respectively; cold crystallization temperature ($T_{cc}$) has not been reported.

### 2.2. Material Conditioning

Given the high sensitivity of the composite material to environmental fluctuations, the filament spool was subjected to a drying cycle at 55 °C for 8 h as recommended by the manufacturer, then stored in a Bambu Lab AMS 2 Pro filament management system [27]. This equipment maintained the storage conditions through an airtight seal combined with active desiccants. Throughout the fabrication process, the filament storage temperature was maintained at 31.44 °C ± 0.49 °C, while the storage humidity was strictly controlled below 5%. This ensured that environmental conditions did not degrade the properties of the material according to the manufacturer’s guidelines.

### 2.3. Specimen Geometry and Fabrication

The tensile testing specimens were designed and fabricated in accordance with ASTM D638 standard type I [28]. The nominal dimensions are in millimeters and the actual printed specimens are detailed in Figure 1.

All specimens were manufactured using a Bambu Lab H2D 3D printer [29], which was continuously fed by the AMS 2 Pro. To process the composite material, the printer’s dual extrusion system was equipped with a 0.4 mm diameter hardened steel nozzle. Furthermore, the machine features an enclosed chamber and a build volume of 325 × 320 × 325 mm. To guarantee optimal first-layer adhesion and prevent warping during fabrication, a textured polyetherimide (PEI)-coated build plate was employed, enhanced by a light coating of a generic spray adhesive.

## 3. Experimental Design and Methodologyy

The overall experimental workflow is illustrated in the diagram of Figure 2. This phase started with the drying cycle of a new PLA-CF filament spool. Then, the tensile specimens were designed using CATIA V5 and processed through Bambu Studio (version 2.4.0.70) to generate the G-code for the Bambu Lab H2D. The manufacturing procedure was conducted in batches of five specimens per build plate; every batch was measured and subjected to the annealing process.

The optimization of the annealing treatment was conducted through an orthogonal experimental design, summarized in Table 3, evaluating temperature and time as the main factors in order to find the combination of levels that maximize the mechanical properties. To clarify the effects of this heat treatment through a wide range of temperature which remains barely documented in the literature for this specific composite, the temperature levels were set approximately 10 °C below the glass transition temperature reported by the manufacturer. Conversely, the maximum level of 95 °C was determined after conducting a rapid scanning of the material’s operative temperatures. This preliminary test revealed that temperatures above 95 °C caused the composite to lose its dimensional stability, making it unusable for engineering applications [24]. The time levels in this investigation were adapted for conventional operative temperatures based on [11], which resulted in a minimum time level of four hours and a maximum of twelve hours. This design yielded 15 parameter combinations, which were tested in batches of five, resulting in a total of 75 evaluated specimens.

The annealing process was performed in a Teralab precision furnace preheated to the desire temperature. The specimens were placed inside in batches of five with the bed surface in contact with the furnace wall. After the designated time, the furnace was turned off and the specimens were allowed to cool inside the chamber for 12 h before mechanical testing. Figure 3 illustrates the schematic process of the annealing heat treatment.

Once the annealing process was completed, the dimensions of the specimens were measured again to record any resulting distortion. These dimensional changes were subsequently quantified as a percentage of deformation relative to the original measurements of the non-annealed specimens.

Subsequently, tensile testing was conducted at a temperature of 24.06 ± 0.78 °C and a relative humidity of 44.93 ± 9.12%, using the INSTRON 600DX universal testing machine (Norwood, MA, USA) [30], equipped with a 250 kN load cell calibrated for polymeric materials, and wedge grips were used. A crosshead speed of 5 mm/min was applied in accordance with the ASTM D638 standard. Strain was calculated based on crosshead displacement, utilizing the integrated Bluehill data acquisition software. To account for machine compliance and ensure accurate strain measurements, the initial distance between the wedge grips was individually recorded for each specimen and used as the effective gauge length.

Following the tensile tests, the SHORE-D hardness of the material was characterized using an E2-D durometer of HOTO INSTRUMENTS (Northbrook, IL, USA) [31]. Direct measurements were taken on the clamping areas of the specimens, evaluating both the top surface and the face directly in contact with the printing bed.

Finally, Differential Scanning Calorimetry (DSC) testing was conducted using a Julia DSC 500 of Anton Paar (Graz, Estiria, Austria) [32], which operates within a range of −35 °C to 500 °C. This equipment features a cooling system for rapid furnace temperature stabilization, a robotic arm that automatically positions the samples in the testing chamber and an inert nitrogen atmosphere. Samples were extracted from the previously tested specimens, with an average mass of 6.52 mg ± 0.24 mg. Aluminum crucibles specifically designed for this analyzer were used. The specific equation to calculate the percentage of crystallinity was based on [33] and is presented in Equation (1).

(1) $$X_{c}=\frac{\Delta H_{m}-\Delta H_{cc}}{\left(1-w\right)\Delta H_{m}^{o}}\times 100$$

where $X_{c}$ is the percentage of crystallinity; $\Delta H_{m}$ is the measured enthalpy at melting; $\Delta H_{cc}$ is the cold crystallization enthalpy; $\Delta H_{m}^{o}$ is the melting enthalpy of the theoretical 100% crystalline PLA, which was set at 93.64 J/g based on [34]; and finally, *w* is the fiber weight fraction, which was established at 0.0688.

### Data Processing and Statistical Analysis

To fully describe the effects of the heat treatment, an Analysis of Variance (ANOVA) and Response Surface Methodology (RSM) were implemented using a custom Python v3.12 script. This approach was enhanced by a backward elimination algorithm designed to iteratively clear the predictive model by removing variables that do not contribute to the description of the phenomenon and only act as noise.

In addition, Young’s modulus was calculated using a piecewise linear regression which identifies the point where the material’s linear behavior ends, allowing for an accurate determination of the initial slope from the experimental scatter data by using the *PiecewiseLinFit* function from the *pwlf* library.

This data processing methodology is similar to the one described in [6], ensuring that only the critical factors governing the annealing process are retained. Filament storage and tensing conditions were excluded from the statistical analysis given that control was maintained. To visualize the complex interactions, RSM plots were generated using the regression equation derived from the refined coefficients.

## 4. Results and Discussion

Following the experimental phase, initial observations revealed a distinct shift in the fracture mechanism driven by the annealing temperature. While the as-printed specimens were completely perpendicular to the load axis fracture characteristic of the mostly aligned short carbon fibers due to the low printing velocity [8], annealing above 75 °C altered the material’s fracture mode into a multi-zone zig-zag pattern, as illustrated in Figure 4a.

Rather than a sudden catastrophic fracture, this behavior suggests a gradual decrease in the material’s load-bearing capacity, generating a characteristic step-like trajectory in the stress–strain curve. As schematically proposed in Figure 4b, this progressive failure mode suggests to be driven by a complex crack deflection mechanism. Based on the existing literature, micro-cracks nucleate at intrinsic stress concentrators such as carbon fiber agglomerations, inter-laminar discontinuities, or random voids inherent to the FDM process [3,35]. As these cracks propagate transversely to the load axis, their trajectory may be obstructed by highly cohesive lamellar crystalline networks induced by the thermal treatment, as reported for similar semicrystalline polymers [34].

Under this interpretation, the crystalline regions could act as barriers due to their superior load-bearing capacity, effectively arresting the transverse crack growth. Consequently, the crack would be forced to deflect toward the path of least resistance, which typically corresponds to the longitudinal adhesion interfaces between the deposited filaments [36,37]. This redirected propagation might continue until the crack encounters subsequent obstacles, such as unaligned carbon fibers or additional reinforced crystalline regions [38]. Ultimately, this proposed constant deflection mechanism prevents a sudden collapse. This suggests that the intact lamellae maintain their ability to transfer the applied stress to the carbon fibers, possibly manifesting as the observed gradual loss of load-bearing capacity [39].

However, from a practical engineering perspective, a component that has lost a significant portion of its maximum load-bearing capacity has sustained irreversible damage and is considered functionally failed. Consequently, to establish a relevant limit, failure is formally defined in this study as the point where the load experiences a 20% drop relative to the UTS. By applying this threshold, the analysis filters out stochastic noise and ensures that reported ductility reflects the true mechanical limits of the material, thereby enabling reliable statistical comparisons such as ANOVA.

This approach is consistent with established practices of polymer mechanics in literature, where a fixed percentage drop from peak load is used to standardize failure definition and appropriately evaluate the functional integrity of the material [40,41].

The ANOVA results for UTS and ductility are summarized in Table 4. This analysis reveals that annealing temperature is the most influential factor for both responses, whereas annealing time does not show a statistically significant influence on these mechanical properties. This behavior is consistent with the findings reported by Kahya et al. [21], who demonstrated that annealing temperature accounts for 81.63% of the UTS variance, while exposure time contributes only 5.95%. Although a formal evaluation of how annealing parameters affect ductility via statistical methods remains underexplored in the current literature, there is a strong consensus that ductility and fracture modes are primarily governed by temperature variations rather than annealing duration in PLA composites [4,42,43].

The ANOVA results for SHORE-D hardness, summarized in Table 5, reveal highly non-linear and complex interactions between the annealing factors. For top surface hardness, time emerges as the most influential parameter, which contrasts with the trends observed for UTS and ductility. This does not exclude annealing temperature from remaining a significant contributing factor. Conversely, bed surface hardness is predominantly governed by the annealing temperature, with thermal exposure time presenting no statistically significant effect on this specific response.

While the current literature generally lacks a rigorous statistical analysis regarding the influence of annealing parameters on these mechanical responses, annealing temperature emerges as the primary driver for overall surface hardness in PLA-CF and other FDM composites [44,45,46].

For Young’s modulus, the resulting ANOVA is summarized in Table 6, where a direct linear relation between both annealing temperature and time emerge. Temperature is the most influential factor in the variation in the material’s rigidity, which is in accordance with the findings of Pandzic et al. [22].

To quantitatively analyze how the evaluated factors modify the material’s mechanical behavior, the predictive regression equations derived from the ANOVA models, summarized in Table 7, were used to generate the RSM plots. These effectively map the composite’s behavior across the entire evaluated range of temperatures and times.

As observed, with the notable exception of Young’s modulus, every mechanical response follows a non-linear trend strongly driven by the annealing temperature, as expressed in Equations (2)–(5) [13,47]. Furthermore, Equation (6) demonstrates that the relationship between Young’s modulus and the thermal factors is strictly linear, suggesting that the composite’s overall rigidity is directly proportional to the enhanced crystallization of the polymer matrix [48,49].

Consequently, the increase in crystallization continuously enhances the material’s rigidity, but properties such as UTS, ductility, and hardness do not follow this linear enhancement. Instead, their maximum values are constrained to a critical intermediate threshold, suggesting a point where material degradation begins.

The resulting optimal parameter levels and their corresponding fit values are summarized in Table 8. To establish statistical reliability, this table includes a Confidence Interval (CI), which indicates the range of the response within which 95% of the tested specimens fall. Furthermore, a Prediction Interval (PI) is also provided, which defines the practical range within which 95% of individual future specimens are expected to perform when subjected to these optimal factors, offering a realistic baseline for manufacturing and design applications.

These results indicate that the absolute mathematical optimum for annealing time is 12 h. However, because exposure time acts as a non-significant factor, varying this parameter between its minimum (4 h) and maximum (12 h) levels at the optimal temperature threshold results in a marginal change of approximately 2% as illustrated in Figure 5. For instance, as predicted by Equation (2), reducing the annealing time from 12 to 4 h at the optimal temperature (80.86 °C) results in a negligible UTS reduction of only 0.46 MPa. This minimal variance opens the opportunity to explore shorter annealing times or to evaluate combinations of higher temperatures with reduced exposure durations to maximize mechanical properties and improve manufacturing efficiency, similar to the approaches reported in [50].

By analyzing the RSM plot for UTS in Figure 5a, a clear optimal region emerges between 75 °C and 85 °C. Specifically, the maximum performance occurs at 80.86 °C, yielding a peak UTS fit value of 59.25 MPa, which represents a 13.74% increase driven by the annealing heat treatment. Conversely, thermal treatment performed below $T_{g}$ fails to induce significant increment, leaving the UTS practically unaltered.

Furthermore, as illustrated in Figure 5b, the ductility contour map closely mirrors the UTS trend, reaching a maximum fit value of 0.0574 mm/mm at 76.82 °C, corresponding to a 24.78% improvement. Interestingly, the material’s ductility exhibits a completely different response when the annealing treatment is applied to neat PLA compared to PLA-CF. Studies such as those by [17,51] indicate that unreinforced PLA tends to experience a reduction in the strain to fracture following heat treatment, as the increase in crystallinity creates a more rigid and brittle matrix.

However, annealing actually promotes ductility in PLA composites [4]. This occurs because the fiber reinforcement acts as a heterogeneous nucleating agent, facilitating a more uniform and controlled crystallization process during thermal exposure [52,53]. The reinforcing phase effectively distributes internal stresses and hinders crack propagation [54], maintaining or even enhancing ductility despite the significant crystallization [55]. Consequently, this heat treatment improves overall mechanical performance without triggering the typical loss of ductility observed in neat PLA.

Regarding SHORE-D hardness, Figure 5c reveals that the theoretical optimal region for the top surface requires annealing just below the glass transition temperature ($T_{g}$), specifically at 55 °C. This condition yields a maximum fit value of 76.06 SHORE-D, which represents a marginal hardness increase of only 1.2%. As reported in similar studies [14], this behavior can be physically attributed to the stress relaxation of the polymer chains induced by the heat treatment, which tends to soften the exposed layers rather than increasing their rigidity.

It is important to consider the thermal boundary conditions during the experimental procedure to fully understand this behavior. The top surface of the specimens was exposed solely to the circulating air inside the chamber, experiencing a slower convective heat transfer. Conversely, the bed surface was placed in direct contact with the metallic furnace walls. This setup subjected the bottom layer to direct and more efficient conductive heat transfer, inevitably creating a localized temperature gradient across the thickness of the component.

This asymmetric thermal exposure may have caused a more accelerated microstructural evolution at the bottom, which resulted in a more pronounced enhancement in the bed surface hardness response, as illustrated by Figure 5d. This figure exhibits a clearly defined optimal region between 75 °C and 85 °C, which aligns with the trends previously described for both UTS and ductility. At exactly 81.67 °C, the model predicts an optimal fit value of 80.68 SHORE-D, representing a 5.58% increase. This contrast highlights that the thermal boundary conditions during post-processing are just as critical as the target temperature itself.

Finally, Figure 5e illustrates the contour plot for the Young’s modulus, which distinctly displays a linear behavior. For this specific response, maximum rigidity is achieved at the highest evaluated temperature (95 °C), yielding a modulus of 4035 MPa, which represents a substantial 39.47% increase.

In order to evaluate the statistical performance of these resulting models, statistical metrics were calculated. These include the standard deviation (SD), which represents the absolute variation in the fitted response; the Coefficient of Variation (CV), which evaluates the stability and repeatability of the data; the Coefficient of Determination ($R^{2}$), which indicates the percentage of the response’s variance that the model can explain; the Adjusted R-squared ($R_{adj}^{2}$), which penalizes the model complexity to prevent mathematical overfitting from non-contributing factors; and the Predictive R-squared ($R_{pred}^{2}$), which evaluates the model’s capacity to described the variance in the behavior of newly untested printed specimens. All of these metrics are summarized in Table 9.

It is noteworthy that, compared to the as-printed material, almost every mechanical response demonstrates high stability during the experimental process, maintaining a CV below 10%. The only exception is Young’s modulus, which exhibits a significantly higher CV and SD, indicating a highly unstable response for the material’s rigidity. This high dispersion can be partially attributed to experimental constraints. The high-capacity load cell utilized may lack the sensitivity required to perfectly capture the whole narrow linear elastic region of the annealed PLA-SCF, introducing data scatter.

Consequently, the predictive capability of the statistical models remains below 65%, particularly for ductility and Young’s modulus. This suggests that the intrinsic stochastic mechanical behavior of AM-manufactured components, compounded by the complex microstructural evolution during annealing, heavily influences the mechanical dispersion. Therefore, in the Young’s modulus and ductility case, rather than serving as exact deterministic tools for component design, the primary utility of these specific models is identifying thermomechanical trends.

In addition to standard performance metrics, Table 10 summarizes the residual diagnostics for the ANOVA models. Normality was evaluated using the Shapiro–Wilk test (where p>0.05 indicates a normal distribution of residuals), and homoscedasticity was assessed via the Breusch–Pagan test (where p>0.05 indicates homoscedasticity). Model structural adequacy was determined using the Lack-of-Fit F-test, where p>0.05 implies that the polynomial complexity is sufficient to describe the data. Finally, influential outliers were evaluated using Cook’s distance, where values greater than 1.0 would indicate isolated data points severely distorting the model’s predictive trend.

As observed, all responses strictly satisfied the homoscedasticity assumption, with the exception of Young’s modulus, which suggests non-uniform variance across the experimental domain, directly corroborating the previously discussed mechanical limitations of the high-capacity load cell when capturing the extremely short linear behavior. Regarding residual normality, only UTS and ductility exhibited minor deviations. However, given the drastic improvements that occur when comparing specimens annealed below the $T_{g}$ to those annealed above 70 °C, an asymmetrical residual distribution is expected. Because the ANOVA framework is fundamentally robust to non-normality provided that homoscedasticity is maintained, the statistical significance of the evaluated factors remains entirely valid.

Regarding the lack of fit, an interesting statistical result emerged. In contrast, the models with the lowest predictive metrics (ductility and Young’s modulus) technically satisfied the lack-of-fit test. This occurred because the inherently high experimental scatter in these stochastic responses statistically hinders any deficiencies in the polynomial fit. Conversely, UTS and hardness models exhibited a significant lack of fit.

Rather than implying the need for a higher-degree polynomial, which could lead to mathematical overfitting, this is a well-known artifact of the high experimental repeatability evidenced by the low CV previously reported. When the error is minor, the F-test becomes hypersensitive; consequently, this lack of fit added by the overall relatively low statistical metrics simply suggests that uncaptured deviations are driven by unmodeled conditions during the process, such as internal furnace humidity, complex convective thermal gradients, or strict cooling rate controls, rather than a flawed regression structure.

Finally, the overall validity of the models was confirmed through Cook’s distance. Across all responses, the maximum value remained well below the critical threshold of 1.0, guaranteeing that no influential outliers are artificially distorting the response surfaces and that the interpolations accurately represent the central tendency of the experimental data.

Nevertheless, as reported by Kumar et al. [56], regardless the inherent variability of FDM composite materials, these results do not invalidate the derived statistical models. Instead, when supported by SD, CI, PI, and RSM, these models remain robust and practical tools for material interpolation and component design.

Subsequentely, the dimensional distortions were quantified by measuring the specimens before and after the annealing treatment. An initial statistical evaluation was conducted to assess the influence of exposure time alongside temperature. However, time demonstrated no statistically significant effect, yielding poor $R^{2}$ values and no clear behavioral trends across the evaluated ranges. This suggests that dimensional distortion in FDM PLA-SCF is primarily governed by the temperature factor. Consequently, for comparison, Figure 6 illustrates the resulting dimensional variations strictly as a function of the annealing temperature, showing longitudinal shrinkage (ΔL), width shrinkage (ΔW), and thickness expansion (ΔTh), represented by the blue, orange, and green lines, respectively.

As observed, these dimensional variations follow a non-linear behavior described by the regression equations provided in the figure. Furthermore, the $R^{2}$ values confirm the high statistical reliability of these trends. The influence of the annealing temperature is severe regarding thickness expansion, yielding a maximum dimensional increase of approximately 12%. In contrast, the thermal treatment induces a comparatively minor longitudinal shrinkage of −4% and a width shrinkage of −5%. Moreover, while width shrinkage remains minimal at temperatures below 70 °C, this contraction accelerates aggressively at temperatures above 80 °C. These thermal deformations result in a net increase of the final cross-sectional area across all evaluated temperatures, which is in accordance with [25]. However, because FDM components are strongly anisotropic, these specific distortion magnitudes depend heavily on the selected raster orientation and infill pattern, suggesting that different geometries would require dedicated dimensional studies.

Regardless of the specific printing configuration, this inherent geometric shift introduces a critical practical trade-off between bulk mechanical performance and dimensional stability. While the thermal treatment markedly improves the macroscopic mechanical response, enhancing strength or ductility depending on the selected thermal window, it inevitably induces a systematic dimensional distortion. Consequently, the appropriateness of annealing is highly application-dependent and must be evaluated against the functional requirements of the component.

For instance, in crash-worthiness and energy-absorbing applications, the targeted performance metric governs the preferred material state: maximizing the Peak Crushing Force typically benefits from higher structural strength, whereas increasing the Mean Crushing Force and overall energy absorption heavily relies on enhanced plastic deformation capacity [57]. For such high-performance structural applications, the bulk mechanical enhancements achieved through annealing far outweigh the dimensional penalties. Furthermore, because this geometric distortion is systematic and predictable, it can be preemptively counteracted by applying inverse scaling factors during the initial CAD or slicing phase. Conversely, if the component’s primary requirement is strictly superficial and does not demand bulk mechanical reinforcement, alternative post-treatments may be considered to improve surface quality with a reduced risk of distortion [58].

To further comprehend the significant enhancement observed in the mechanical properties, DSC testing was conducted. The resulting thermograms are illustrated in Figure 7, where the as-printed baseline is consistently represented in blue for comparative purposes.

Interestingly, in DSC, the $T_{g}$ is typically described as a step change in heat capacity rather than a sharp endothermic peak [59,60]. However, in the highly amorphous as-printed specimen, this thermal transition manifests as a pronounced peak. This occurs because the response of 3D-printed polymers at $T_{g}$ depends heavily on how far the glassy structure is from equilibrium. Because the FDM process inherently yields in thermal residual stresses due to rapid cooling rates and sharp temperature gradients, the polymer remains in a highly non-equilibrium state. During DSC reheating, the sample rapidly releases this stored energy through enthalpy recovery, causing the transition event to appear abrupt [61,62].

In contrast, specimens that have already undergone an annealing treatment have successfully relaxed these internal microstructural stresses and the newly formed crystalline domains act as barriers that constrain the mobility of the remaining amorphous chains, resulting in a significantly smoother $T_{g}$ transition [63,64].

Furthermore, annealed specimens lack a distinct $T_{cc}$ peak, strongly indicating that the heat treatment successfully induced matrix crystallization. Instead, the thermograms display local variations between 125 °C and 135 °C. This is a well-documented phenomenon attributed to a later recrystallization process [65]. Imperfect crystals formed during annealing require less energy to melt than fully developed regions. As the temperature rises during the DSC scan, these imperfect structures briefly melt by absorbing energy and immediately reorganize into more stable crystalline lamellae by releasing energy, generating these local peaks. Subsequently, a prominent and final endothermic melting peak emerges at $T_{m}$, representing the complete fusion of all crystalline structures within the specimen.

The data acquired from thermographs is summarized in Table 11. For the as-printed specimens, $T_{g}$ could be established at 65.69 °C, which is 2.69 °C higher than indicated by the manufacturer’s data sheet. Then, a distinct $T_{cc}$ emerges at 109.3 °C with a $\Delta H_{cc}$ of 9.56 J/g. Finally, the $T_{m}$ occurs at 148.2 °C, which is 16.8 °C lower than the reported baseline, yielding a $\Delta H_{m}$ of 11.67 J/g. Based on these data, the $X_{c}$ of the as-printed composite is 2.43%.

Regarding the annealed specimens, a substantial increase in $X_{c}$ is evident. The minimum was observed at test 7 (75 °C for 4 h) and the maximum was achieved at test 15 (95 °C for 12 h) which is nine and twelve times the baseline, respectively. The resulting enhancement exhibits a proportional relationship with the increase in both annealing temperature and exposure time. However, the variation in $T_{g}$ does not follow a predictable trend, reaching its peak value in test 9 (75 °C for 12 h).

In contrast, the $T_{m}$ remains remarkably stable across all conditions, exhibiting a variation of only 2.9%. Consequently, the overall increase in crystallization is primarily dictated by the final enthalpy values, which heavily depend on annealing temperature rather than time.

As observed, although the thermally induced crystalline domains grow proportionally with temperature and linearly enhance Young’s modulus, this increase in $X_{c}$ exhibits a highly non-linear influence on the other mechanical responses. As Bharat et al. [35] describe, a delicate balance exists between promoting polymer chain diffusion across the composite’s distinct layers and inducing matrix crystallization. While chain diffusion requires a certain degree of mobility to effectively weld adjacent layers, the progressive crystallization of the matrix inherently restricts this movement. Based on the mechanical responses, it is suggested that excessive or highly accelerated crystallization rigidly locks the polymer chains before adequate interlayer diffusion occurs. Under these conditions, the matrix becomes highly rigid, but the poorly welded interfaces act as severe stress concentrators. This explains why higher crystallinity does not yield a monotonic improvement for UTS and ductility. Beyond the optimal thermal threshold, the composite becomes overly brittle, and the weak interlayer bonding triggers premature failure. Consequently, it is critical to identify the exact optimal window of annealing temperature to provide sufficient mobility for chain diffusion to strengthen layer bonding, while simultaneously crystallizing the matrix to maximize the overall thermomechanical benefits.

It is crucial to acknowledge that the microstructural mechanisms discussed in this study, including crack deflection pathways, interlayer bonding dynamics, fiber agglomeration effects, and matrix–fiber interface changes, are phenomenological interpretations derived from thermal analysis (DSC) and macroscopic mechanical data, supported by the existing literature. While DSC robustly validates the phase transitions associated with annealing, it inherently lacks direct topographical or crystallographic resolution. Therefore, the proposed microstructural interactions should be considered as highly plausible hypotheses rather than directly observed phenomena.

In order to confirm these mechanisms, future work must incorporate advanced microstructural characterization. Specifically, Field Emission Scanning Electron Microscopy will be essential to visually verify fiber pull-out, interfacial debonding, and exact crack trajectories. Furthermore, techniques such as X-ray Diffraction to independently quantify crystalline structures and Atomic Force Microscopy (AFM) or Raman spectroscopy to evaluate molecular alterations at the interfaces are proposed for future investigations.

For the experimental validation stage, a batch of five specimens was tested under annealing parameters established at 80 °C for 12 h based on the previous results. This temperature was deliberately chosen because it maximizes UTS while substantially increasing ductility, Young’s modulus, and bed surface hardness without inducing significant top surface softening. At this thermal level, the linearly interpolated $X_{c}$ reaches approximately 24.12%.

Regarding the 12 h exposure period, this duration was selected for the verification run simply because it represented the mathematical maximum for property enhancement in the optimization algorithms. However, as demonstrated by the ANOVA results, the statistical effect of exposure time is marginal. Therefore, this study does not prescribe a single optimal condition. Instead, the generated data enables engineers to make informed, criterion-based decisions. Depending on specific design requirements and manufacturing constraints, shorter annealing times can be selected with confidence, provided the temperature is strictly controlled, as this will yield very similar mechanical improvements.

The resulting stress–strain curves from this validation test are illustrated in Figure 8. Table 12 summarizes the validation results for UTS, ductility, and Young’s modulus. The model for UTS consistently matches the experimental data, yielding a maximum relative error of only 3.47% and maintaining all values within the previously established PI. Conversely, ductility exhibits less consistent behavior, resulting in a relative error of 19.65% and an outlier that differs from the respective PI (0.0745 mm/mm), which was an expected deviation that was previously anticipated by its low $R_{\left(pred\right)}^{2}$ and higher CV.

Similarly, Young’s modulus presents the highest relative error at 29.1%. As discussed earlier, this deviation can be primarily attributed to the high-capacity load cell utilized, which lacked the extreme sensitivity required to accurately capture the narrow linear zone of this composite, thereby introducing data scatter and noise specially in this mechanical response.

Regarding surface properties, the SHORE-D hardness validation results are detailed in Table 13. As anticipated by their low CV, these remain the most consistent and stable mechanical responses evaluated in this study. The maximum errors were remarkably low, at just 1.31% for the top surface and 1.16% for the bed surface, both being inside the PI previously established.

Despite the previously discussed statistical limitations and lack of fit, the mechanical responses demonstrated remarkable stability. The validation tests confirmed that the experimental results fall consistently within the previously established PI, proving that these models are valuable and reliable tools for engineering design. A notable exception is Young’s modulus, where the statistical metrics were adversely affected by mechanical noise from the load cell’s resolution limits. Nevertheless, the trend captured by the model is strongly supported by the physical evidence obtained through DSC testing. Similarly, the predictability of ductility is severely hindered by the intrinsically stochastic fracture behavior of 3D-printed composites, particularly due to random void distribution and complex crack nucleation mechanisms. Consequently, the surface mappings for rigidity and ductility should be interpreted as robust qualitative trends rather than strictly deterministic models.

Finally, Table 14 summarizes the dimensional distortion results. Considering that maintaining tight geometric tolerances is one of the most prominent challenges in the FDM process, the recorded absolute errors are considerably low. Longitudinal shrinkage (ΔL) yielded the highest absolute deviation among the three measurements at 10.94%, followed by thickness expansion (ΔTh) at 9.96%, and a minor error of 7.29% for width shrinkage (ΔW). Overall, these results provide a highly predictable and reliable margin for calculating the expected thermal distortion of mechanical components subjected to this specific manufacturing and annealing regime.

## 5. Conclusions

This research investigated the effects of annealing heat treatments on the mechanical enhancement of 3D-printed PLA-SCF composite based on a baseline of optimized printing parameters in order to fully develop the effects of the annealing heat treatment. Overall, these findings are in strong agreement with the recent literature, and we obtained the following conclusions:

- Annealing temperature is the dominant factor driving the variation in nearly all evaluated mechanical responses. Conversely, the annealing time yields a marginal impact on the overall mechanical enhancement of the material. Although the statistical certainty of temperature dominance for Young’s modulus was affected by the load cell resolution limits inducing null homoscedasticity, the DSC analysis corroborates that temperature, rather than time, dictates the stiffness enhancement due to the significative increase in crystallinity.
- Dimensional distortion exhibits a non-linear behavior. Thermal deformation is most severe in the thickness direction, yielding an expansion of 12%, while the longitudinal and width directions experience comparatively minor shrinkage of −4% and −5%, respectively.
- The optimal threshold for the PLA-SCF falls within the 75 °C to 85 °C window for 12 h, effectively enhancing the material’s mechanical properties. However, due to the statistical non-significance of the annealing time, lower time levels can be used to obtain very similar results to the maximums. Rather than prescribing a single rigid parameter, this range represents a critical engineering trade-off between maximizing mechanical property gains and mitigating dimensional distortions. This information enables engineers to select specific time and temperature parameters based on their own design criteria, constraints, and manufacturing needs.
- Compared to the parametrically optimized 3D-printed state, UTS improved by 13.74% (59.25 MPa) at 80.86 °C, ductility increased by 24.78% (0.0574 mm/mm) at 76.82 °C, bed surface hardness by 5.58% (80.68 SHORE-D) at 81.67 °C and Young’s modulus scaled linearly, reaching a maximum 39.47% increment (4035 MPa) at the highest tested temperature of 95 °C. In contrast, top surface hardness experienced a detrimental effect, softening due to stress relaxation.
- DSC analysis confirmed a substantial increase in crystallinity, which is proportional related to the enhancement of the material’s overall stiffness. Conversely, the remaining mechanical properties exhibit a highly non-linear trend governed by a delicate balance between polymer chain diffusion and the progressive recrystallization of the matrix.

Despite these contributions, future work should prioritize advanced microstructural characterization using scanning electron microscopy and X-ray diffraction to obtain direct evidence of morphological evolution, fracture-surface features, and potential changes in crystallinity and phase structure. In parallel, systematic parametric studies varying fiber volume fraction, fiber-length distribution, and PLA grade are recommended to evaluate the generalizability of the observed trends, while the mechanical assessment should be expanded beyond quasi-static testing to include fatigue, creep, impact, and environmental aging protocols in order to quantify long-term durability and performance.

## Figures and Tables

**Figure 1:**
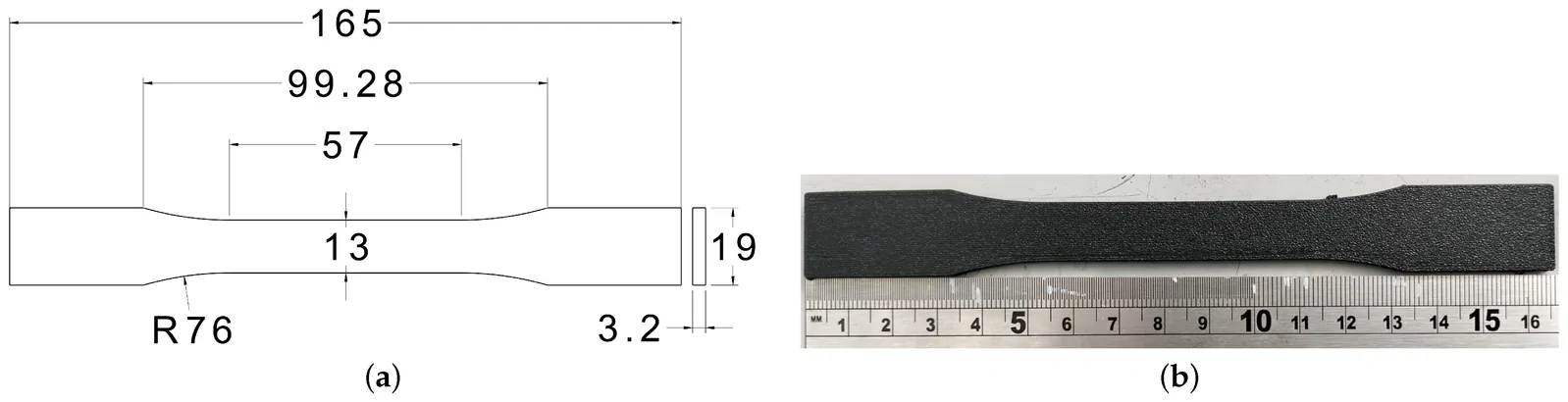
Testing specimens. (**a**) Nominal dimensions of the ASTM D638 Type I specimen and (**b**) printed specimen.

**Figure 2:**
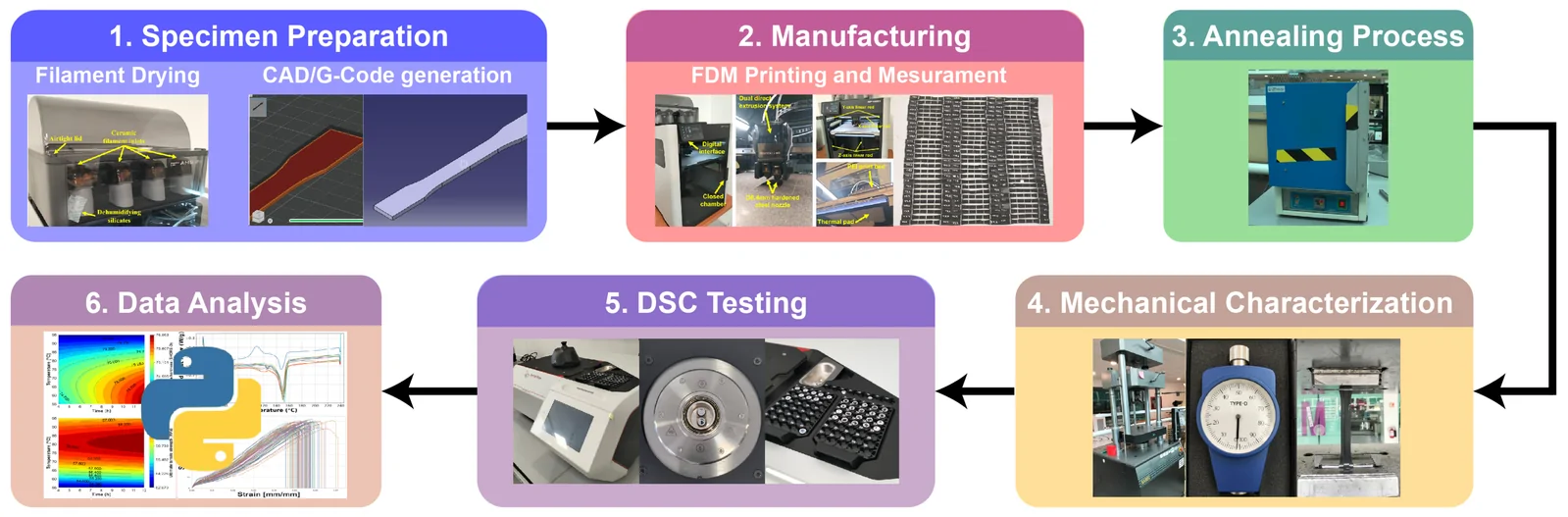
Experimental workflow methodology.

**Figure 3:**
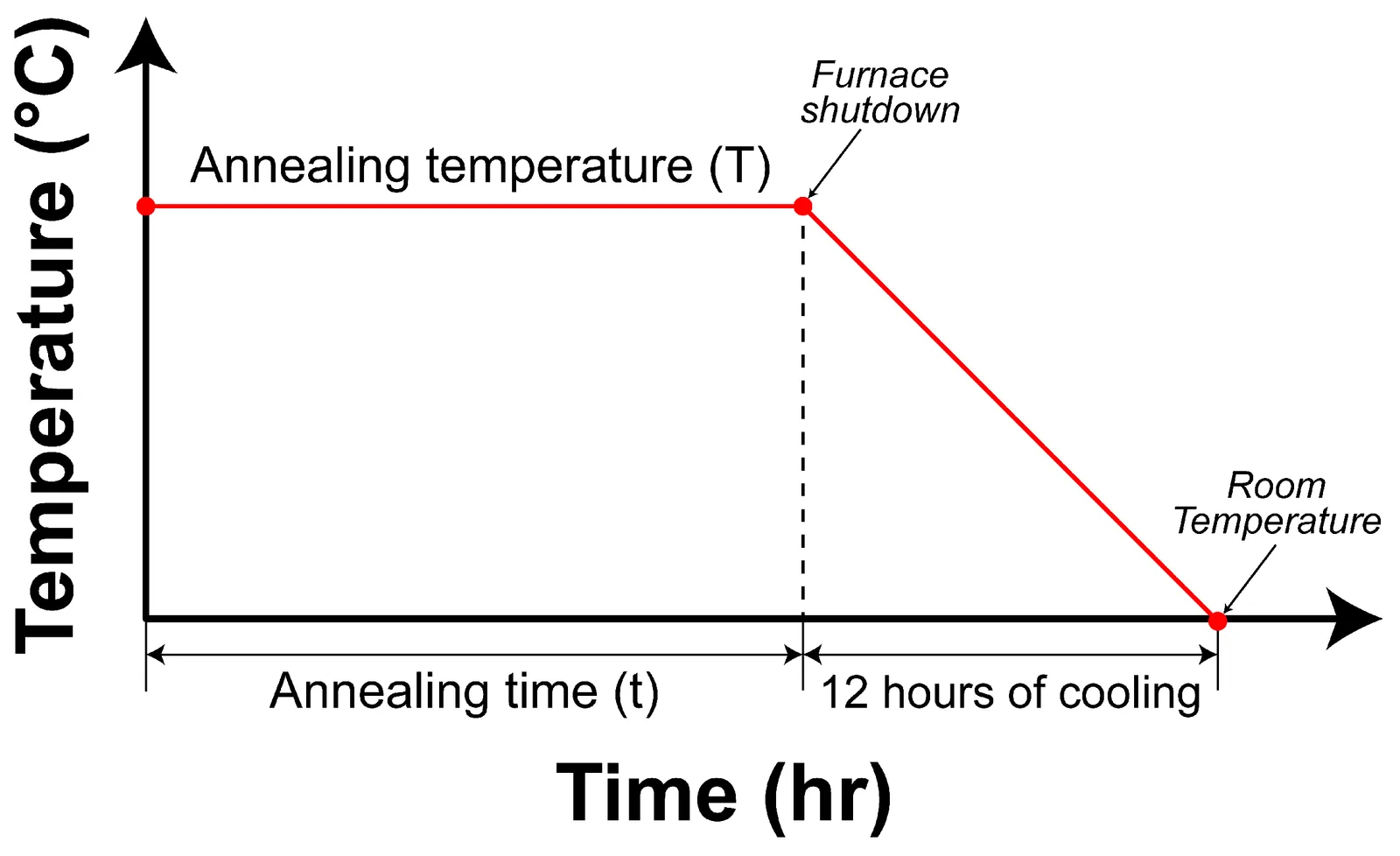
Schematic process of the annealing heat treatment.

**Figure 4:**
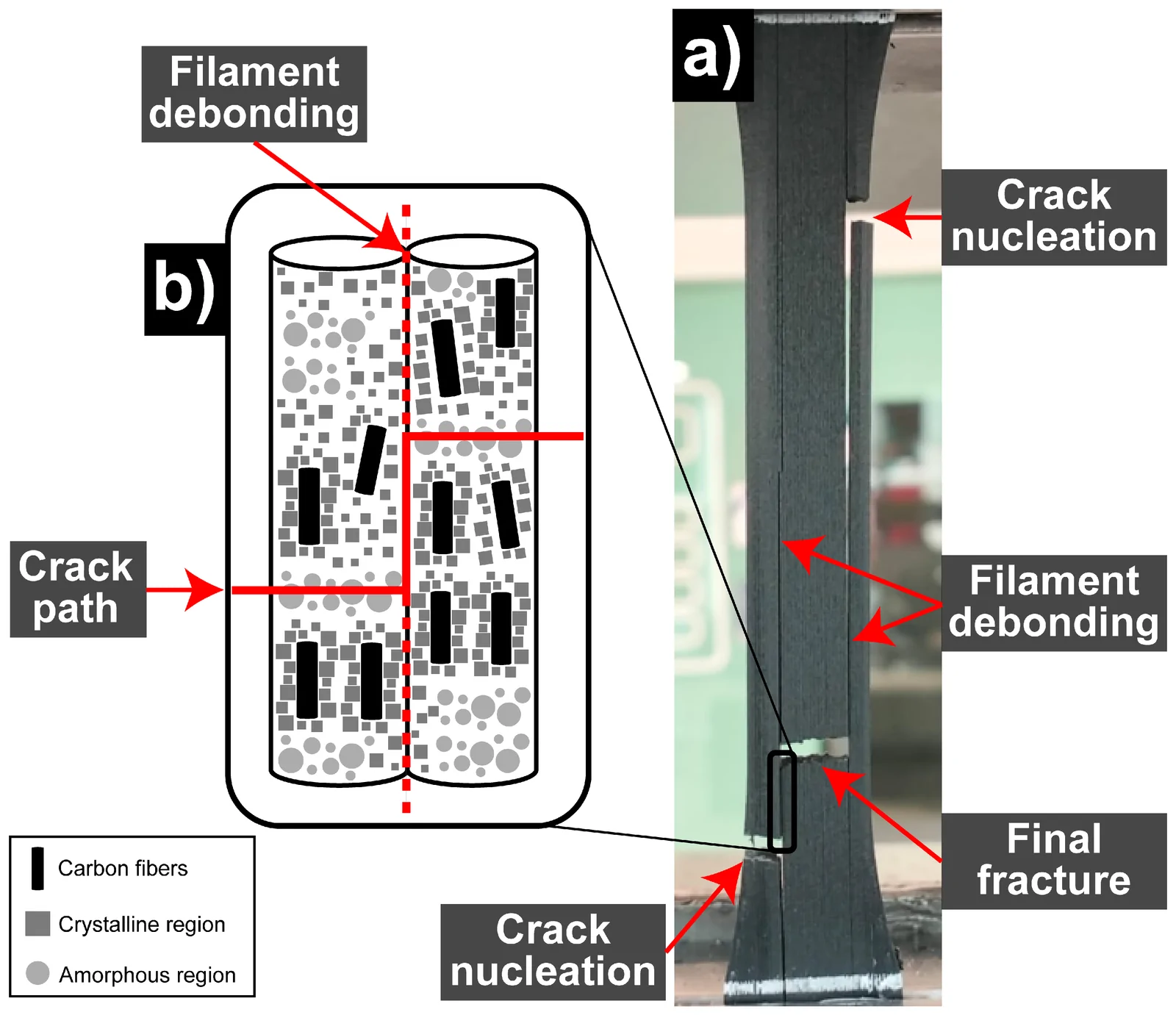
Multi-zone zig-zag fracture in the real annealed specimen and the schematic process of the fracture.

**Figure 5:**
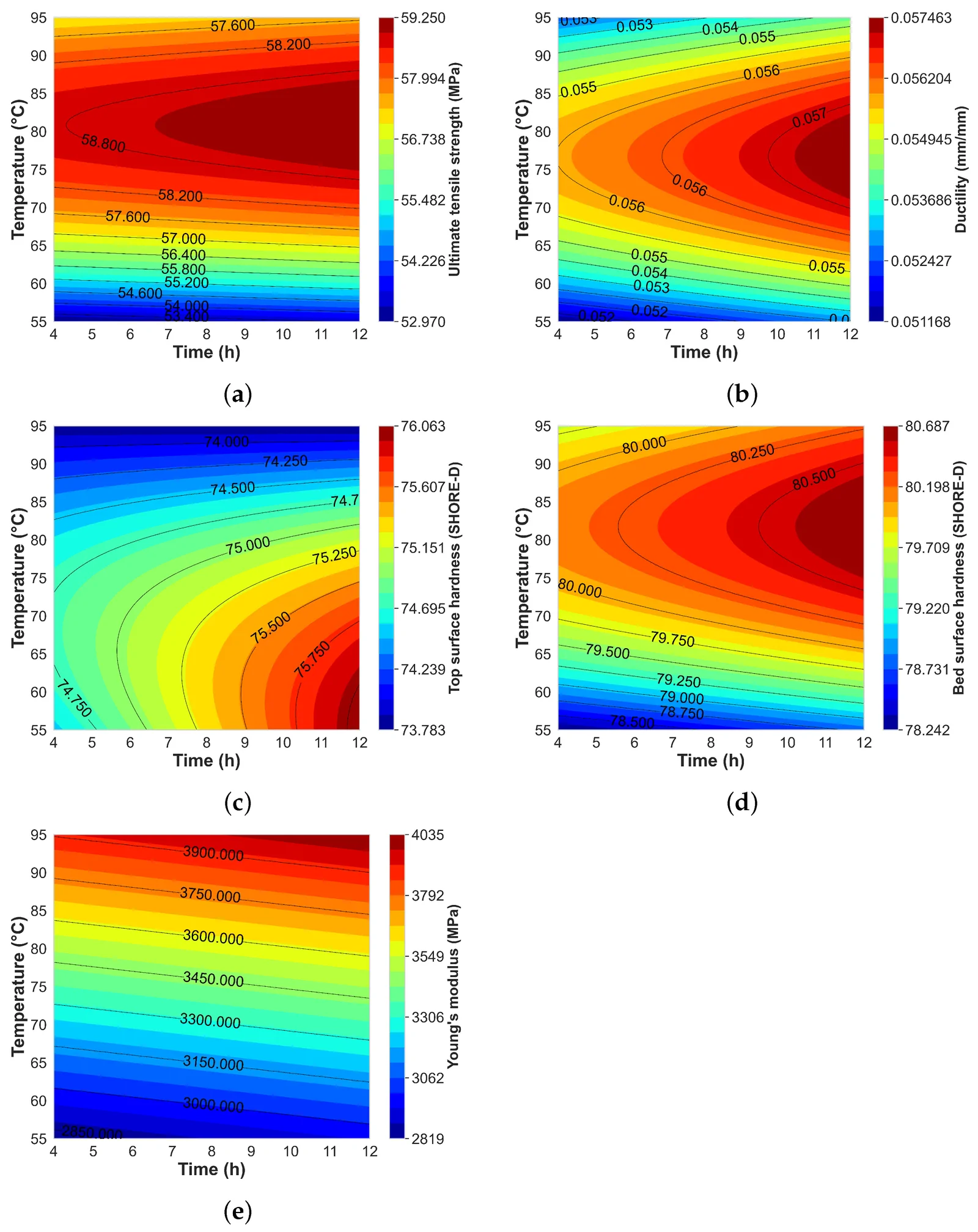
RSM plots of the main factors’ interactions.

**Figure 6:**
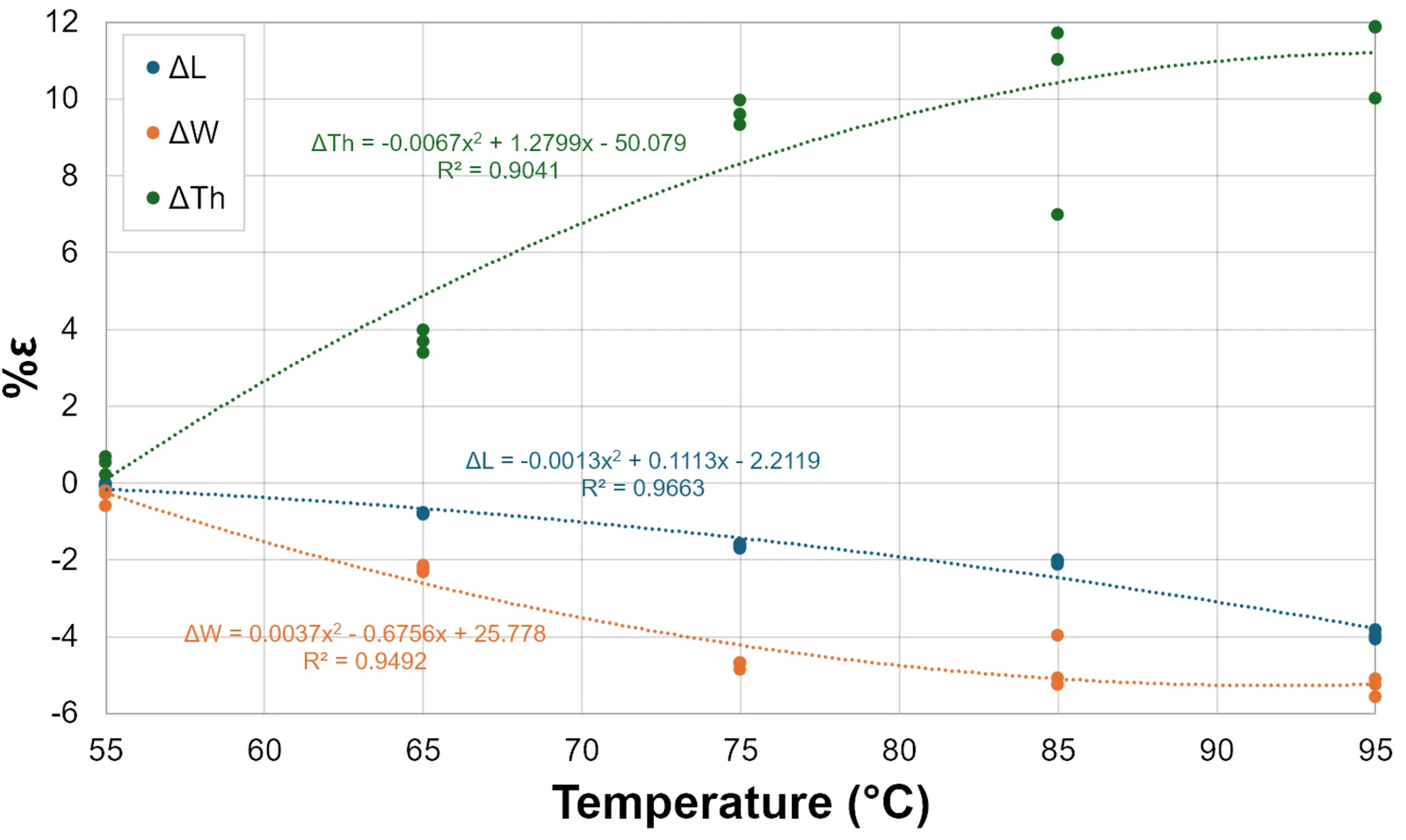
Dimensional changes induced by different levels of annealing temperatures.

**Figure 7:**
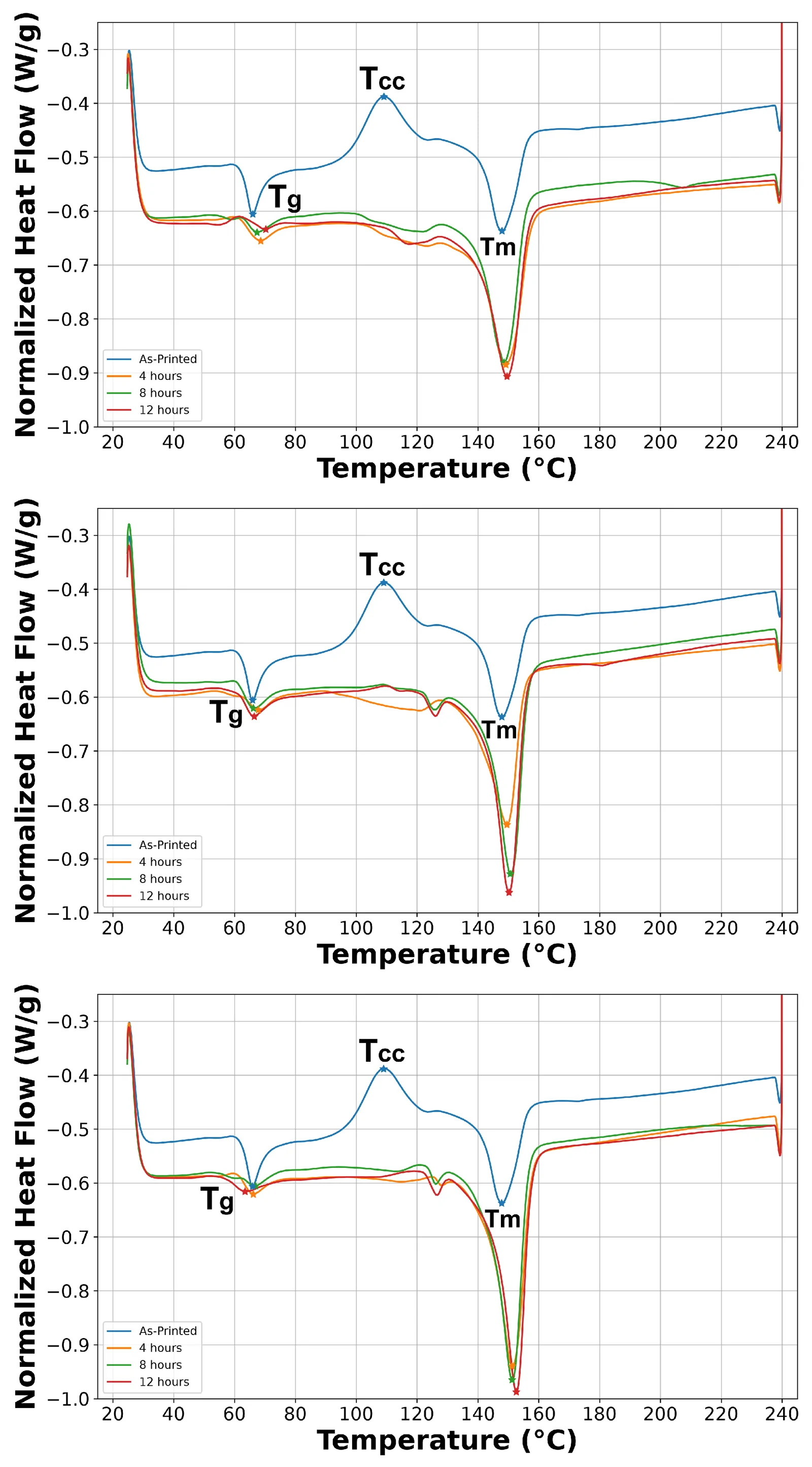
Differential Scanning Calorimetry plots of (**top**) 75 °C, (**middle**) 85 °C and (**bottom**) 95 °C annealed specimens.

**Figure 8:**
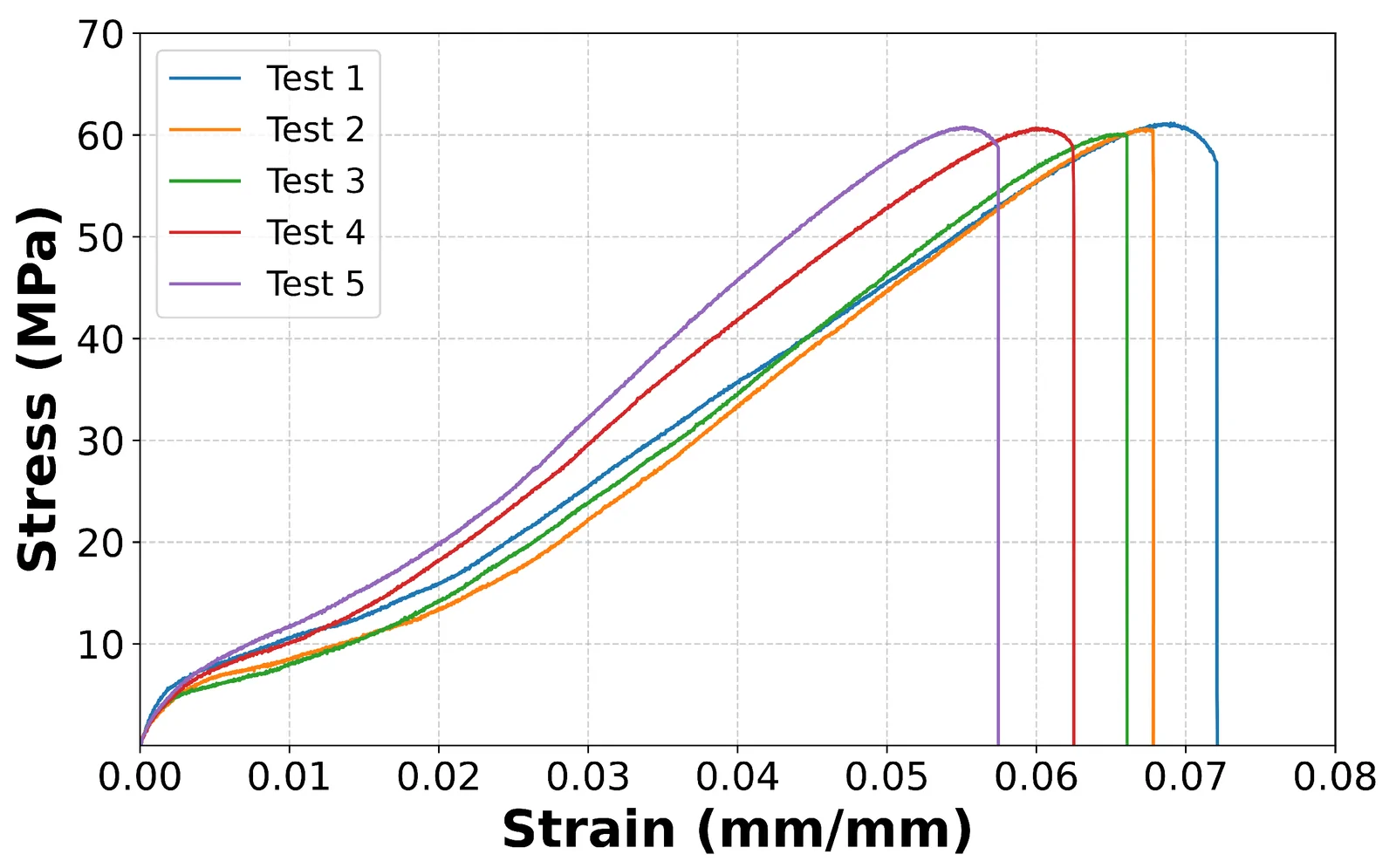
Stress–strain curves of the validation testing.

**Table 1:** Printing and conditioning specifications for test specimens.

Parameter	Value or Description
Layer height	0.08 mm
Printing speed	20 mm/s
Nozzle temperature	240 °C
Bed temperature	45 °C
Chamber temperature	30 °C
Infill pattern	Rectilinear
Infill percentage	100%
Raster orientation	0°
Number of walls	2
Nozzle diameter	0.4 mm
Specimen build orientation	Flat (XY plane)
Cooling	Post-print ambient cooling on bed
Removal procedure	Manual peel-off from bulid plate
Filament drying	55 °C for 8 h using Bambu Lab AMS

**Table 2:** Mechanical properties of PLA-CF based on [6,26].

Properties	Non Optimized Printing Parameters	Optimized Printing Parameters
Young’s Modulus	2160 ± 120 MPa	2893 ± 305 MPa
Tensile Ultimate Strength	38 ± 4 MPa	52.09 ± 1.57 MPa
Elongation at break	8.4 ± 3.2%	4.6 ± 0.37%
Top Surface Hardness	Not reported	75.16 ± 1.3 SHORE-D
Bed Surface Hardness	Not reported	77.38 ± 1.01 SHORE-D

**Table 3:** Experimental design for the annealing optimization.

Test	Temperature (°C)	Time (h)
1	55	4
2	55	8
3	55	12
4	65	4
5	65	8
6	65	12
7	75	4
8	75	8
9	75	12
10	85	4
11	85	8
12	85	12
13	95	4
14	95	8
15	95	12

**Table 4:** ANOVA model for ultimate tensile strength and ductility.

	Ultimate Tensile Strenght					Ductility				
	DF	Adj SS	Adj MS	F	*p*	DF	Adj SS	Adj MS	F	*p*
**Intercept**	1	0.197267523	0.197267523	0.063588453	0.8016401439	1	5.72151 $\times \;10^{-7}$	5.72151 $\times \;10^{-7}$	0.023824181	0.877771033
**T**	1	183.495804	183.495804	59.14919016	0.0000000001	1	0.000210942	0.000210942	8.783578804	0.00413459
**t**	1	2.744878893	2.744878893	0.884801505	0.3500793361	1	3.4015 $\times \;10^{-5}$	3.4015 $\times \;10^{-5}$	1.416374911	0.237965261
**T^2^**	1	158.8929608	158.8929608	51.21855513	0.0000000006	1	0.000202708	0.000202708	8.440697007	0.00488898
**Residual**	71	220.2600247	3.102253868			71	0.001705104	2.40155 $\times \;10^{-5}$		

**Table 5:** ANOVA model for SHORE-D hardness.

	Top Surface Hardness					Bed Surface Hardness				
	DF	Adj SS	Adj MS	F	*p*	DF	Adj SS	Adj MS	F	*p*
**Intercept**	1	264.597199	264.597199	492.8980557	2.08552 $\times \;10^{-33}$	1	266.3902359	266.3902359	478.6687512	2.81852 $\times \;10^{-33}$
**T**	1	3.829148017	3.829148017	7.133029449	0.009403224	1	17.50647363	17.50647363	31.45686569	3.66534 $\times \;10^{-07}$
**t**	1	5.755469528	5.755469528	10.72142771	0.001647887	1	3.6992	3.6992	6.646983282	0.01200767
**T × t**	1	3.949714286	3.949714286	7.357623209	0.008397419					
**T^2^**	1	3.7636	3.7636	7.01092502	0.010002401	1	14.82685714	14.82685714	26.64194192	2.13672 $\times \;10^{-6}$
**Residual**	70	37.57735238	0.53681932			71	39.51314286	0.556523139		

**Table 6:** ANOVA model for Young’s modulus.

	Young’s Modulus				
	DF	Adj SS	Adj MS	F	*p*
**Intercept**	1	3,386,363	3,386,363	4.859952	0.030683
**T**	1	11,091,308	11,091,308	15.91773	0.000157
**t**	1	206,625.9	206,625.9	0.29654	0.587742
**Residual**	72	50,168,844	696,789.5		

**Table 7:** Resulting equations from the ANOVA models.

Response	Equation	
Ultimate Tensile Strength	$-0.0087T^{2}+1.4065T+0.0585t+1.69$	(2)
Ductility	$-9.82\times 10^{-6}T^{2}+0.0015T+0.0002t-0.0028$	(3)
Top Surface Hardness	$-0.0013T^{2}+0.207T+0.458t-0.005\left(T\times t\right)+66.54$	(4)
Bed Surface Hardness	$-0.0026T^{2}+0.4344T+0.068t+62.113$	(5)
Young’s Modulus	27.192T+16.071t+1259.34	(6)

**Table 8:** Optimal parameter combination and values calculated using the ANOVA model.

Response	T (°C)	t (h)	FIT ± SD	95% CI Lower	95% CI Upper	95% PI Lower	95% PI Upper
Ultimate Strength	80.86	12	59.25 ± 1.76 MPa	58.48 MPa	60.02 MPa	55.65 MPa	62.84 MPa
Ductility	76.82	12	0.0574 ± 0.005 mm/mm	0.0552 mm/mm	0.0597 mm/mm	0.0474 mm/mm	0.0674 mm/mm
Top Surface Hardness	55	12	76.06 ± 0.73 SHORE-D	75.55 SHORE-D	76.56 SHORE-D	74.51 SHORE-D	77.6 SHORE-D
Bed Surface Hardness	81.67	12	80.68 ± 0.74 SHORE-D	80.36 SHORE-D	81.01 SHORE-D	79.16 SHORE-D	82.2 SHORE-D
Young’s Modulus	95	12	4035 ± 834 MPa	3628 MPa	4443 MPa	2322 MPa	5749 MPa

**Table 9:** Performance summary of the ANOVA models.

Response	CV (%)	$R^{2}$ (%)	$R_{\left(\mathrm{adj}\right)}^{2}$ (%)	$R_{\left(\mathrm{pred}\right)}^{2}$ (%)
Ultimate Strength	3.1	59	57.26	54.7
Ductility	8.97	12.98	9.30	2.41
Top Surface Hardness	0.98	48.92	46	40.4
Bed Surface Hardness	0.94	48.91	46.75	42.76
Young’s Modulus	24.36	18.38	16.11	11.71

**Table 10:** Residual diagnostic results for each mechanical response.

Response	Homoscedasticity (*p*-Value)	Normality (*p*-Value)	Lack-of-Fit (*p*-Value)	Maximum Outlier (Cook’s Distance)
Ultimate Tensile Strength	0.851	0.012	<0.001	0.126
Ductility	0.202	0.004	0.061	0.325
Top Surface Hardness	0.840	0.505	0.029	0.230
Bed Surface Hardness	0.855	0.242	<0.001	0.084
Young’s Modulus	0.057	0.508	0.27	0.120

**Table 11:** Differential Scanning Calorimetry results.

Test	$T_{g}$ (°C)	$T_{\mathrm{cc}}$ (°C)	$T_{m}$ (°C)	$\Delta H_{\mathrm{cc}}$ (J/g)	$\Delta H_{m}$ (J/g)	$X_{c}$ (%)
As-Printed	65.69	109.3	148.2	9.56	11.67	2.434
7	67.88		149.14		19.766	22.804
8	67.1		149.78		19.92	22.982
9	70.03		149.72		20.912	24.126
10	65.89		149.65		18.537	21.386
11	65.55		150.89		22.118	25.518
12	65.95		150.28		22.755	26.252
13	67.75		151.47		24.164	27.878
14	65.93		151.5		24.504	28.271
15	62.85		152.6		25.528	29.452

**Table 12:** Results of the validation test for UTS, ductility and Young’s modulus models.

Test	UTS (Mpa)			Ductility (mm/mm)			Young’s Modulus (Mpa)		
	Fit Value	Experimental Value	Absolute Error (%)	Fit Value	Experimental Value	Absolute Error (%)	Fit Value	Experimental Value	Absolute Error (%)
1	59.25	61.31	3.47	0.06227098	0.0745	19.65	3655	4168	14.1
2		60.58	2.24		0.0679	8.96		4007	9.7
3		60.18	1.57		0.0661	6.12		3936	7.7
4		60.73	2.49		0.0625	0.36		3519	3.7
5		60.88	2.76		0.0574	7.79		4718	29.1

**Table 13:** Results of the validation test for SHORE-D hardness.

Test	Top Surface Hardness (SHORE-D)			Bed Surface Hardness (SHORE-D)		
	Fit Value	Experimental Value	Absolute Error (%)	Fit Value	Experimental Value	Absolute Error (%)
1	75.41	76.4	1.31	81.05	81.8	0.92
2		75.4	0.02		81	0.07
3		75.2	0.28		80.8	0.32
4		76.4	1.31		82	1.16
5		76.2	1.04		82	1.16

**Table 14:** Results of the validation test for the dimensional changes.

Test	ΔL (%)			ΔW (%)			ΔTh (%)		
	Fit Value	Experimental Value	Absolute Error	Fit Value	Experimental Value	Absolute Error	Fit Value	Experimental Value	Absolute Error
1	−1.726	−1.85	7.02	−4.67	-5.01	7.29	9.63	9.57	0.72
2		−1.91	10.94		−4.75	1.71		8.67	9.96
3		−1.88	9.12		−4.57	2.05		9.32	3.21
4		−1.90	10.21		−4.73	1.38		8.80	8.63
5		−1.87	8.39		−4.74	1.47		9.64	0.02

## Data Availability

The authors confirm that the data supporting the findings of this study are available within the article.
